# The Three Bacterial Lines of Defense against Antimicrobial Agents

**DOI:** 10.3390/ijms160921711

**Published:** 2015-09-09

**Authors:** Gang Zhou, Qing-Shan Shi, Xiao-Mo Huang, Xiao-Bao Xie

**Affiliations:** 1 Guangdong Institute of Microbiology, Guangzhou 510070, Guangdong, China; E-Mails: zgbees@gdim.cn (G.Z.); xmhuang@gdim.cn (X.-M.H.); xiaobaoxie@126.com (X.-B.X.); 2 State Key Laboratory of Applied Microbiology Southern China, Guangzhou 510070, Guangdong, China; 3 Guangdong Provincial Key Laboratory of Microbial Culture Collection and Application, Guangzhou 510070, Guangdong, China

**Keywords:** antimicrobial agents, defense lines, resistance mechanism, action sites, new theory

## Abstract

Antimicrobial agents target a range of extra- and/or intracellular loci from cytoplasmic wall to membrane, intracellular enzymes and genetic materials. Meanwhile, many resistance mechanisms employed by bacteria to counter antimicrobial agents have been found and reported in the past decades. Based on their spatially distinct sites of action and distribution of location, antimicrobial resistance mechanisms of bacteria were categorized into three groups, coined the three lines of bacterial defense in this review. The first line of defense is biofilms, which can be formed by most bacteria to overcome the action of antimicrobial agents. In addition, some other bacteria employ the second line of defense, the cell wall, cell membrane, and encased efflux pumps. When antimicrobial agents permeate the first two lines of defense and finally reach the cytoplasm, many bacteria will make use of the third line of defense, including alterations of intracellular materials and gene regulation to protect themselves from harm by bactericides. The presented three lines of defense theory will help us to understand the bacterial resistance mechanisms against antimicrobial agents and design efficient strategies to overcome these resistances.

## 1. Introduction

Antimicrobial agents such as antibiotics, disinfectants and preservatives have been widely used to control or kill microorganisms in the past and will continue to be in long-term use [1,2,3]. Generally, all natural, semi-synthetic or synthetic substances with capacity of slowing or inhibiting the growth and reproduction of microorganisms and even killing them can be regarded as antimicrobial agents. These agents exhibit a specific action mechanism whereby microbial metabolism and physiological processes are modified including translation, DNA replication and cell wall biosynthesis [4,5]. Correspondingly, various biological and molecular responses of bacteria may be developed in the presence of antimicrobial agents [6]. Based on published literatures and the guidelines of Clinical and Laboratory Standards Institute (CLSI), antimicrobial resistance can be defined as an ability of microorganisms to resist the effects of one or more antimicrobial agents that they are originally sensitive to [7]. The emergence of antimicrobial resistance has become a major threat to public health and even causes huge losses in agriculture and industry around the world [8,9]. Better understanding of the resistance mechanisms of bacteria to antimicrobial agents can therefore guide the use of existing agents with improved activity and develop more efficient new ones. Many resistance mechanisms for bacteria to combat antimicrobial agents have been found in the past decades [10,11,12,13,14]. General resistance mechanisms include alterations of target sites, limited diffusions or impermeabilities, enzymatic modifications, efflux pumps and genetic adaptations [11,15]. Moreover, different resistance mechanisms will be employed by one given bacterium to protect themselves from one given antimicrobial agent or different ones [16].

**Figure 1:**
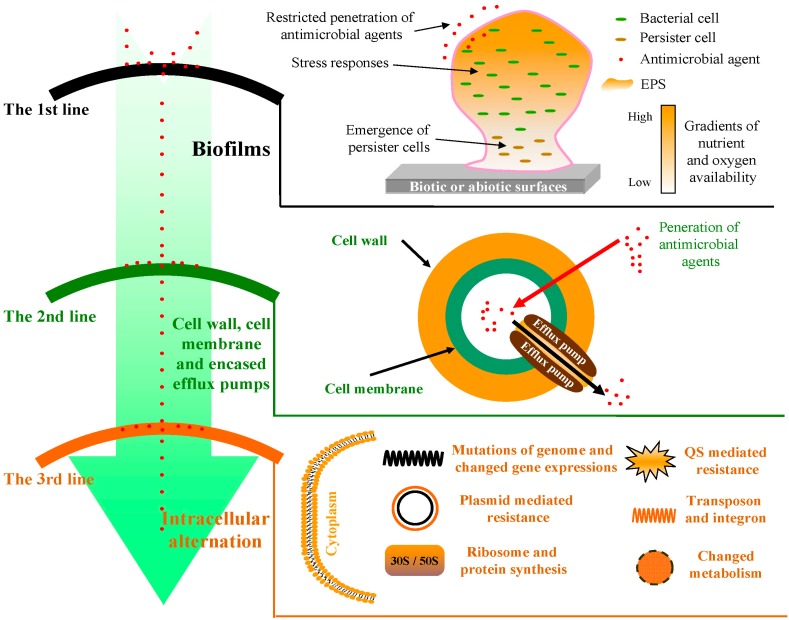
Simplified diagram summarizing the mechanisms of the three defense lines of resistance to antimicrobial agents for bacteria. There are three lines of defense for bacterial cells to overcome death by antimicrobial agents. The first defense line is bacterial biofilms, which limits the penetration of antimicrobial agents. The cell wall, cell membrane and the encased efflux pumps construct the second defense line to limit the absorbance or elevate the excretion of antimicrobial agents. When antimicrobial agents enter the bacterial cells, they meet the third line of defense, involving the alteration of target sites, regulation of gene expression and production of certain enzymes.

Bacterial resistance mechanisms can be categorized into different groups based on different criteria. For example, antimicrobial resistances can be classified into two broad groups according to the acquired modes of resistance for bacteria to antimicrobial agents, *i.e.*, intrinsic and acquired [5,17]. In this review, we attempt to address the general mechanisms that underlie the development of bacterial resistances to antimicrobial agents and categorize bacterial resistance mechanisms into three groups (namely three lines of defense) according to their sites of action of resistance (Figure 1): the first line of defense is bacterial biofilms; the cell wall, cell membrane and encased efflux pumps consist of the second line of defense; and, when bactericides ultimately get into the bacterial cells, intracellular biochemistry and genetic responses play an important role in resistances and are considered as the third line of defense. Developing these defense line theories will help us to more clearly understand the main resistance mechanisms of bacteria to overcome antimicrobial agents.

## 2. The First Line of Defense: Bacterial Biofilms

Biofilms are defined as a thin layer of microbial communities adhered to each other on organic or inorganic surfaces and enclosed by their secreted matrices of extracellular polymeric substance (EPS) [18,19]. It has been accepted that microorganisms live as a mode of biofilms but not solitary entities during most of its microbial life [20]. Biofilms formation can trigger serious environmental problems such as biofouling and dozens of human infections such as cystic fibrosis and urinary catheter cystitis [20,21,22]. When bacterial cells attach to a solid biotic or abiotic surface, they will gradually produce hydrated EPS and finally form the typical spatial structures of biofilms [13,23,24]. Typical biofilms development includes several stages, from the initial attachment of bacterial cells to maturation, as well as the final dispersion of biofilms [18,25]. Biofilms formation not only provides a protected mode of growth for bacterial cells to survive in hostile environments but also increase resistance level of bacteria to antimicrobial agents [26]. Although several mechanisms have been proposed to explain increased resistance to antimicrobial agents in bacterial biofilms, it is becoming obvious that only a combination of multi-factorial mechanisms or a collective resistance mechanism could interpret these resistances observed in biofilms communities [23,27].

### 2.1. Restricted Penetration of Antimicrobial Agents

Compared with planktonic cells, one of the distinguishing characteristics of biofilms is the production of EPS [24,28]. The EPS matrix of biofilms can limit and even prevent the transport of antimicrobial agents to the cells by either reacting with the bactericides, or sorption, or electrostatic and hydrophobic interactions, or size exclusion, or degradation of biocides [29,30]. The binding of antimicrobial agents to the protective EPS matrix will delay the time for antimicrobial agents to reach bacterial cells, resulting in the increased resistance level observed in biofilms [7,31]. As measured by a chlorine-detecting microelectrode, a commonly used disinfectant of chlorine did not reach higher than one fifth of the bulk media’s concentration within a mixed biofilms formed by *Klebsiella pneumoniae* and *Pseudomonas aeruginosa* [32]. The penetration rates of amikacin and gentamicin were slower when penetrating through *P. aeruginosa* biofilm than piperacillin and imipenem [33]. Meanwhile, it has been observed that thick biofilms presented as a more efficient barrier than thin biofilms in delaying the penetration of antimicrobial agents [34].

Although the binding of antimicrobial agents to the possible reaction sites present in the EPS of biofilms can limit the transport of antimicrobials, unhindered delivery to the cells would resume when all sites in the matrix have been bound [23]. It has been reported that the biofilms formed by *Staphylococcus epidermidis* allow for the diffusion of rifampicin and vancomycin across the membrane, which implies that these antibiotics can efficiently penetrate biofilms [35]. More recent research has revealed that the biofilms of *Burkholderia pseudomallei* played a role as a diffusion barrier for ceftazidime and imipenem but not for trimethoprim or sulfamethoxazole [36]. The above evidence implies that the resistance mechanisms of biofilms to antimicrobial agents cannot be completely explained by the limitation of diffusion by EPS, but it does represent an initial barrier that can delay antimicrobial penetration.

### 2.2. Physiological Gradients

Microscale gradients in nutrient concentrations or growth factors are another well-known feature of biofilms [37]. There is a possible anaerobic condition in the microenvironment of biofilms because oxygen concentration is limited in the center of biofilms compared with at the surface [18]. The response of bacterial cells located in different places within a biofilm community to antimicrobial agents can greatly vary [38]. When bacterial cells grow in biofilms, growth will slow or stop due to a limited nutrient environment, which is generally accompanied by an increase in resistance to antimicrobial agents [21]. Only the upper metabolically active cells in *P. aeruginosa* biofilms could be effectively killed by fluoroquinolones and tetracycline [39]. On the contrary, the deeper slow-growing cells but not the actively growing cells that acquired adaptive resistance mediated by the pmr operon and the *mexAB-oprM* genes could be eradicated by an antimicrobial compound of lipopeptide colistin [39]. Because of limited nutrient availability, protein synthesis and metabolic activity in biofilms are also stratified, which is another explanation for the increased resistance of biofilms to antimicrobial agents [40,41]. In addition, Evans and colleagues reported that there were significant differences in resistance to ciprofloxacin between *P. aeruginosa* biofilms and planktonic cells at fast growth rates but not at slow growth rates, which indicated that only a reduction in growth rate could not completely explain all resistance mechanisms in biofilms to antimicrobial agents [42].

### 2.3. Persistence

Any given cells in biofilms live in a highly protected phenotypic state, grow at a different rate, and differentiate similar to spore formation [43]. A spore-like biofilm cell state (namely persistence) contributes significantly to the reduced susceptibility of biofilms to antimicrobial agents and provides a powerful genetic explanation for the resistance mechanisms in biofilms [43,44,45]. Indeed, it has been reported that the most significant resistance mechanism in *S. epidermidis* biofilms is that there are a large number of persister subpopulations in its biofilms [45]. Meanwhile, most of the population in biphasic biofilms is rapidly killed but a fraction of the cells are unaffected even by prolonged antibiotic treatment which is in support of the above hypothesis of persistence [46]. Persister cells cannot be affected by inhibitory concentrations of antimicrobial agents, and exhibit the ability to overcome stressful conditions, likely due to transcriptional programming [44].

### 2.4. General Stress Response

An efficient stress response system has been constructed by bacterial cells to deal with harmful environmental conditions [47]. These adaptive responses also contribute to resistance mechanisms in biofilms. Bacteria utilize a distinct subfamily of extracytoplasmic function (ECF) sigma factors to regulate extra cytoplasmic function and to serve as bacterial transcriptional regulators in the response to various stresses [48,49]. The ECF mutants (PGN_0274 and PGN_1740) of *Porphyromonas gingivalis* strain 33277 had increased biofilm formation compared with the wild-type [50]. Similar results were also found in a previous study conducted by another group. Inactivation of ECF-10 encoded by PP4553 in *Pseudomonas putida* KT2440 resulted in enhanced formation of biofilms after 24 h of incubation and two- to four-fold increased antibiotic resistance to quinolone, β-lactam, sulfonamide, and chloramphenicol antibiotics [51]. A comparative transcriptomic analysis was performed to identify differentially expressed genes during biofilms growth of *P. aeruginosa* [52]. The results showed that regulons associated with Anr-mediated hypoxia stress, RpoS-regulated stationary phase growth, and osmotic stresses were up-regulated in the biofilms and significantly enriched. The abilities of mutant strains deficient in *rpoS*, *relAspoT*, or *anr* to form biofilms were reduced when exposed to ciprofloxacin. These results suggested that multiple genes controlled by overlapping starvation or stress responses contributed to the protection of *P. aeruginosa* biofilms from ciprofloxacin [52].

As shown above, there are complex resistance mechanisms in bacterial biofilms so that it is quite difficult to diminish or eradicate them. However, some traditional and novel approaches used to control biofilms have been proposed in the past decades [53]. Combinations of tobramycin and clarithromycin have shown reasonable success in clearing *P. aeruginosa* pulmonary biofilm infections [54]. Meanwhile, combinations of peptide 1018 and some antibiotics such as ciprofloxacin decreased the concentration of antibiotic required to decrease initial biofilms formation and trigger cell death in mature biofilms. These findings suggested that treatment with the peptide represented a novel strategy to potentiate antibiotic activity against initial and mature biofilms formed by multidrug-resistant pathogens [55]. In addition to antibiotics, a variety of non-chemical substances were also developed to combat bacterial biofilms. D-amino acid can be used to inhibit biofilm formation by *Staphylococcus aureus* or *P. aeruginosa* through causing the release of amyloid fibers that linked cells in the biofilm together [56]. Based on a better understanding of the genetic basis of biofilm formation and development, conserved intracellular signals and regulators might be manipulated and used to control biofilms [57]. A great number of gene manipulation methods have been employed to induce biofilms dispersal [58,59,60]. More detailed approaches or strategies to combat bacterial biofilms could be found in the corresponding reviews [57,61]. Furthermore, we believe that more and more physiological, biochemical or molecular methods can be exploited to conquer biofilms and its resistances.

## 3. The Second Line of Defense: Bacterial Cell Wall and Cell Membrane

In order to exert their antibacterial activity, a wide variety of antimicrobial agents must attain a sufficiently high concentration at intracellular target sites. In order to reach their target site(s), they have to traverse the bacterial cell wall and membrane, which are crucial for maintaining cell shape and exchanging nutrients or signaling molecules. At the same time, the cell wall and membrane are also important targets for many antimicrobial compounds, including β-lactams, glycopeptides, fosfomycin, daptomycin, polymyxin, and ionophore antibiotics. A change in cell wall or membrane conformations or limited penetration of antimicrobial agents through these two physical barriers may induce the emergence of resistance. Moreover, restricting access or efficiently removing the antimicrobial agents by efflux pumps that are encased in the cell wall and membrane also contribute to increased levels of resistance. Hence, the cell wall, membrane and encased efflux pumps constitute the second line of defense for bacteria in combating antimicrobial agents.

### 3.1. Cell Wall

The bacterial cell wall is responsible for the maintenance of cell shape as well as other important functions [62,63]. Compared with parental cell walls, the peptidoglycan of a highly vancomycin-resistant mutant of *S. aureus* exhibited a significantly lower degree of cross-linkage. This observation and the results of vancomycin-binding studies suggested that alterations in the structural organization of the mutant cell walls blocked access of the vancomycin molecules to wall biosynthesis sites [64]. Resistance to glycopeptides such as vancomycin and teicoplanin derives from the synthesis of abnormal pentapeptide precursors, where precursors posses altered termini (e.g., d-Ala-d-lactate or d-Ala-d-ser) and a lower affinity for glycopeptides [65]. β-Lactams can block the transpeptidase and transglycosylase cross-linking enzymes in the peptidoglycan layer of cell walls [66]. Bacteria make use of two main strategies for protection against β-lactams: alteration in Penicillin-Binding Proteins (PBPs), which reduces the affinity of β-lactams for action sites, and production of β-lactamases, which hydrolyzes the ring of β-lactams rendering the molecule inactive [66,67,68,69].

### 3.2. Cell Membrane

Around the inside of the cell wall is a bacterial cell membrane, which works as a selective filter that allows or restricts cell permeation of substances, including antimicrobial agents, in or out of the cell. Membrane-active antimicrobial agents include multi-targeted lipopeptide daptomycin, peptidic antibiotics such as colistin and polymyxin B, and the ionophore antibiotics monensin and salinomycin [70]. The outer cell membrane of *P. aeruginosa* presents a significant permeability barrier to the penetration and excluding of antimicrobial molecules, which can lead to the occurrence of bacterial resistance [71]. Small hydrophilic antibiotics such as β-lactams and quinolones can only cross the outer membrane by passing through the aqueous channels provided by porin proteins. An outer membrane protein (OMP, 35 kDa) was found in wild-type (WT) cells of *P. aeruginosa* but not in isothiazolone-resistant cells. Therefore, it was proposed that this protein was the channel utilized by isothiazolone to transverse the cell membrane [72]. Furthermore, it was also found that each of the methylchloroisothiazolone (MCI)-resistant isolates of *P. aeruginosa* lacked a 42 kDa protein, which is believed to be a porin known as OprD when compared with MCI-sensitive isolates. These findings reveal that the outer membrane can act as a permeability barrier, allowing for MCI resistance [73]. In addition, there were also some differences in OMP profiles between an isothiazolone-resistant strain of *Burkholderia cepacia* BC-IR induced from WT, and an isolated strain of *B. cepacia* BC-327 separated from industrial contamination samples [74] suggesting that different bacteria obtained from different sources may make use of different OMPs to exhibit their resistances.

### 3.3. Multi-Drug Efflux Pumps

Bacterial efflux systems are able to transport a wide variety of antimicrobial agents with different structures conferring multi-drug resistance (MDR) [75]. Generally, bacterial efflux pumps have been classified into two groups based on the energy source used by the pump [76,77,78]. The primary group includes the ATP (adenosine triphosphate)-binding cassette (ABC) super-family which uses the energy of ATP binding and hydrolysis for efflux; the secondary group includes the multidrug and toxic compound extrusion (MATE) family, the major facilitator super-family (MFS), the resistance-nodulation-division (RND) family, and the small multidrug resistance (SMR) family, all of which use the energy of the electrochemical potential of the membrane to power efflux [75,78,79,80]. Except for the RND super-family, which is only found in Gram-negative bacteria, efflux systems of the other four families are widely distributed in both Gram-positive and -negative bacteria [81]. It has been observed that the resistance of many bacteria to antimicrobial agents mainly developed by means of activation of efflux pumps [82]. The AcrAB/TolC system, comprised of an inner membrane transporter AcrB, an outer membrane protein channel TolC, and a periplasmic adaptor protein AcrA, is one of the most well-characterized efflux system in Gram-negative bacteria such as *Escherichia coli* [83,84]. Meanwhile, the compositional stoichiometry of this pump is 3:6:3 (AcrB:AcrA:TolC) [85]. When AcrAB/TolC system is activated, the linker protein AcrA firstly fold on itself resulting in close contact of the AcrB and TolC proteins. Then, an exit path is provided from the inside to the outside of the cell so that antimicrobial agents can be pumped out through this channel [83,86,87]. More recently, a novel G288D substitution in AcrB of standard *E. coli* and *Salmonella* strains was found to have contributed to the resistance to ciprofloxacin [88]. In addition, four multidrug RND efflux systems of *mexAB*-*oprM*, *mexCD-OprJ*, *mexEF*-*oprN*, and *mexXY*-*oprM* are significant for clinically relevant resistance in *P. aeruginosa* [78,89,90]. Each above efflux system has its substrate specificities for antimicrobial agents. For example, extrusion of aminoglycosides and a group of the β-lactams is specific to MexXY-OprM and MexAB-OprM, respectively [91,92]. Furthermore, MexCD-OprJ can extrude novobiocin, cefsulodin, and flomoxef [91]. The up-regulation of MexCD-OprJ correlates with an increased resistance to ciprofloxacin, cefepime, chloramphenicol, or norfloxacin in *P. aeruginosa* [93,94]. In summary, efflux pump proteins contribute to the intrinsic and acquired resistance of *P. aeruginosa* through the multidrug active efflux process [90,95]. Besides the well-studied efflux pumps above, new pumps, such as KexD in *Klebsiella pneumoniae* [96] and MdeA in *Streptococcus mutans* [97], continue to be reported in the past few years. Taken together, the advances in understanding of efflux pumps and their resistance mechanisms will help us to propose a promising strategy and design efflux pump inhibitors for tackling multidrug resistance in bacteria [78,98]. Several natural and synthetic efflux pump inhibitors have been evaluated and are shown to reduce resistance in some studies. An inhibitor of RND transporters of 3,4-dibromopyrrole-2,5-dione decreased the MICs of seven antibiotics between 2- and 16-fold in over-expressing three archetype RND transporters (AcrAB-TolC, MexAB-OprM, and MexXY-OprM) strains [99]. Several naturally occurring indole alkaloids such as α-yohimbine and its derivatives showed efflux pumps inhibitory potential and reduced MIC of tetracycline up to eight folds against a multidrug resistant clinical isolate of *E. coli* MDREC-KG4 [100].

In addition, efflux pumps have been also reported as one of the factors contributing to biofilms resistance to antimicrobial agents in several bacteria such as *P. aeruginosa* and *E. coli* [77]. Mutant strains of *P. aeruginosa* without the novel efflux pumps PA14 and PA1874 to PA1877 (PA1874-1877) become more sensitive to tobramycin, gentamicin and ciprofloxacin, specifically when these strains are grown as biofilms, which also provides an explanation for why these genes were important for biofilm resistance to antibiotics [101]. Deletion of the 16 operons encoding RND type efflux pumps demonstrated that RND-3, RND-8 and RND-9 protected biofilms against tobramycin in *Burkholderia cenocepacia* J2315 [102]. A more recently research has also demonstrated that efflux pumps contribute to glutaraldehyde resistance in *P. fluorescens* and *P. aeruginosa* biofilms based on RNA-Seq analysis and chemical inhibition assay [103]. However, some authors also found that the efflux pumps mentioned above did not contribute to the antibiotic-resistant phenotype and ciprofloxacin resistance in *P. aeruginosa* and *E. coli* biofilms, respectively [104,105], suggesting that there are other resistance mechanisms in the biofilms of *P. aeruginosa* and *E. coli*.

## 4. The Third Line of Defense: Intracellular Alteration

Although there are two lines of defense outside of the bacterial cells as described above, a variety of antimicrobial agents can still successfully penetrate into the cells and exert their activity. Inside the cells, antimicrobial agents may inhibit bacterial growth or kill bacteria through destroying metabolic systems and regular gene expressions. In response, bacterial cells will do their best to compete with antimicrobial agents including employing strategies like the alteration of target sites, production of antagonistic agents and regulation of gene expressions. All of the resistant strategies occurring inside of bacterial cells are considered as the third line of defense.

### 4.1. Bacterial Ribosome and Protein Synthesis

Proteins within bacterial cells carry out a myriad of vital cell functions like catalyzing enzymatic reactions, sensing and passing on signals and making important physical structures. As such, inhibition of protein biosynthesis may lead to the death of bacteria. Antimicrobial agents target bacterial protein synthesis usually through interacting with ribosome and inhibiting its function. For example, the association of aminoacyl-tRNA with the bacterial 30S ribosomal subunit can be prevented by tetracyclines resulting in the inhibition of bacterial protein synthesis [106,107]. Resistance to tetracyclines can also occur partly through the production of ribosomal protection proteins (RPPs) [108]. Aminoglycosides, such as streptomycin, can also bind to the 30S bacterial subunit of ribosomes leading to inhibition of protein synthesis and final occurrence of resistance [109,110]. Moreover, the target site (*i.e.*, ribosomal) mutations were also contributed to the resistance to aminoglycosides [111]. In contrast, chloramphenicol acts by binding to the 50S ribosomal subunit to inhibit protein synthesis. Resistance to chloramphenicol has been described in part by the presence of chloramphenicol acetyltransferase (CAT), an enzyme that inactivates the drug [112]. The mode of action of macrolides is that they can bind to the bacterial 50S ribosomal subunit thus inhibiting protein synthesis. The most common resistance mechanism to macrolides arises from methylation of an adenine residue in domain V of the 23S rRNA [113,114].

### 4.2. Metabolic Pathway

Bacteria may also develop resistance to an antimicrobial agent by increasing the production of a metabolite, which competes with the active site of the agents. It has been reported that *S. aureus* develops resistance to sulfonamide, an analogue to paraamino-benzoic acid (PABA), by increasing the production of PABA that would competitively displace sulfonamide [5]. This unusual increased production of PABA has been found to be due to mutations in the regulatory gene of the phosphate biosynthetic pathway [5].

### 4.3. Quorum Sensing (QS) Systems

One of significant mechanisms for bacterial communities to rapidly receive input from the environment and coordinately elicit an appropriate response under the stresses of antimicrobial agents is QS systems whose signaling requires an *N*-acyl-homoserine lactone (acyl-HSL) synthase (I protein) and a transcription factor with acyl-HSL-dependent activity (R protein) [115]. A signaling molecule served as an indicator of the population density would activate transcription of the genes encoding the R and I proteins under the help of the R protein-acyl-HSL complex so that a positive feedback regulatory mechanism was created [116]. In *P. aeruginosa*, a pathogenic QS bacterium, the transcription of genes encoding virulence factors would be also induced by activated R proteins in QS systems which signaling pathway become a target for the design of small molecule inhibitors [117,118]. A QS-deficient *lasR rhlR* mutant of *P. aeruginosa* is more sensitive to H_2_O_2_ because of the less production of catalase and NADPH-producing dehydrogenases [119].

Moreover, QS systems also contribute to the resistance mechanisms of biofilms. Two QS systems of LasR-LasI and RhlR-RhlI are global regulators of gene expression in the opportunistic pathogen *P. aeruginosa* [120]. A *lasR*–*lasI* mutant of *P. aeruginosa* lost its ability to form a biofilm with normal architecture, while *lasI* mutant biofilms exhibited increased sensitivity to sodium dodecyl sulfate (SDS) [121]. Similarly, *P. aeruginosa* biofilms with *lasI* and *rhlI* mutations display increased susceptibility to kanamycin compared to WT biofilms [122]. Meanwhile, the use of QS inhibitors (QSI) has been proposed as a potential antibiofilm strategy [123,124].

### 4.4. Genetic Regulation

#### 4.4.1. DNA Synthesis

Resistance to quinolones has been a problem ever since these drugs were introduced into clinical medicine. Quinolones are considered as inhibitors of the essential bacterial enzymes, DNA gyrase and DNA topoisomerase IV resulting in inhibition of DNA replication [125]. Similarly, chromosomal mutations in the subunits of DNA gyrase and topoisomerase IV also lead to the emergence of fluoroquinolones resistance [126].

#### 4.4.2. RNA Synthesis

Rifampicin (Rif), a member of rifamycin family, can inhibit the bacterial RNA polymerase (RNAP) and is broadly used as an anti-tuberculosis agent to control bacterial pathogens. RNAP isolated from a Rif-resistant mutant of *Mycobacterium smegmatis* is less sensitive to Rif *in vitro* than WT strains, confirming that one mechanism of Rif resistance in mycobacteria is through alteration of RNAP [127]. Combined with biochemical analysis, the crystal structure of core RNAP complexed with Rif in *Thermus aquaticus* demonstrated that the path of the elongating RNA was directly blocked by Rif when the transcript becomes 2 to 3 nucleotide (nt) in length [128]. More recent literature has also shown that mutations in the *rpoB* gene encoding for the β-subunit of RNAP mainly contribute for Rif resistance [129]. Furthermore, resistance to aminoglycosides was also found to be related to RNA synthesis. Among Gram-negative pathogens such as *Enterobacteriaceae* and glucose-nonfermentative microbes, methylation of 16S ribosomal RNA (rRNA) mediated by a newly recognized group of 16S rRNA methylases has recently emerged as a new resistance mechanism against aminoglycosides [130].

#### 4.4.3. Plasmids

A plasmid is a small, circular and double-stranded DNA molecule in bacterial cells. It is physically separated from chromosomal DNA and can replicate independently. Generally, plasmids can give the bacteria some survival advantages under certain conditions, such as the ability to survive in the presence of an antimicrobial agent [5]. A great number of enzymes encoded by plasmids can catalyze antimicrobial agents into a non-toxic form resulting in resistances. It has been found that the penicillinase plasmids, severed as a series of extrachromosomal resistance factors, carry determinants of resistance to penicillin and, in some cases, erythromycin in *S. aureus* [131]. Likely, it has been reported that heavy metal and formaldehyde resistance are mediated by enzymes, which are also encoded by corresponding plasmids [132]. Formaldehyde can be detoxified and reduced by a plasmid-encoded and constitutively expressed NAD^+^-gluthathione-dependent dehydrogenase in *P. aeruginosa* and *P. putida* [133]. Methylation of specific nucleotides in rRNA by methylases, such as erythromycin ribosome methylase (*ermC*) [134,135] and chloramphenicol–florfenicol resistance (*cfr*) methyltransferase [136], which are carried on plasmids [137], to protect the drug-binding sites is one of the important means by which bacteria achieve resistance to several antimicrobial agents including macrolides, lincosamines, erythromycin, phenicols, pleuromutilins, and streptogramins [138]. The activity of β-lactamase encoded by plasmids usually induces high-level resistance of bacteria to broad-spectrum β-lactam antibiotics [139]. Both topoisomerase IV and DNA gyrase can be bound by pentapeptide repeat proteins (PRPs) encoded by plasmid-encoded quinolone resistance genes of *qnr* group to protect the bacterial cells from the lethal action of quinolones [140,141,142]. The plasmid-borne fluoroquinolone-resistance determinants of *qnr* genes were widespreaded in *Enterobacteriaceae*. LexA is the central regulator of the SOS response to DNA damage. A LexA-binding site was found in the promoter region of all *qnrB* alleles and *qnrB2* expression is regulated through the SOS response in a LexA/RecA-dependent manner [143]. Besides of the above evidences, the proteins or enzymes derived from plasmids also play a role at other defense lines. An IncH1 plasmid in a strain of *Citrobacter freundii* was found to encode a novel tripartite resistance nodulation division (RND) pump [144].

#### 4.4.4. Chromosome

As we all know, the genetic information is stored in bacterial chromosome. Mutations of the target gene in bacterial chromosome can lead to the emergence of antibiotic resistance [145,146,147]. Many *E. coli* resistant strains from patients with uncomplicated urinary tract infections had mutations in *gyrA*, *parC*, *parE*, *marOR*, or *acrR* [148]. The SOS mutagenic response can be induced in the presence of non-lethal concentration of antimicrobial agents such as quinolones resulting in the emergence of resistance in *E. coli* [149]. In addition, resistance genes can horizontal transfer between two bacteria through transduction, conjugation and transformation so that the received strain gets the feature of resistance [146]. It has been demonstrated that comparative genomic analysis of *S. suis* strains with diverse drug-resistant phenotypes provided evidence that horizontal gene transfer is an important evolutionary force in shaping the genome of multi-drug-resistant strain of *S. suis* R61 [150]. Moreover, the horizontal dissemination of antibiotic resistance genes can be promoted by SOS response [151].

Transposon (also called jumping gene) is a genetic element that can move from one location in a chromosome to another location in the same or a different chromosome and thus alter the genetic constitution of the organism [152]. Many researchers have reported that transposon is also involved in bacterial resistance mechanisms. A Tn3-related transposon of Tn1546 confers glycopeptide resistance by synthesis of depsipeptide peptidoglycan precursors in *Enterococcus faecium* BM4147 [153]. Besides of Tn1546, other transposons including Tn1547, Tn1549, Tn916, conjugative transposons of the Tn916/Tn1545 family, Tn21 and a group of closely related transposons (the Tn21 family), also confer bacteria to resistant antimicrobial agents such as vancomycin and tetracycline [154,155,156,157,158].

Integrons are genetic units characterized by their ability to capture and incorporate gene cassettes by site-specific recombination resulting in antibiotics resistances [159,160,161,162]. All integrons are usually composed of three key elements necessary for the capture of exogenous genes: an *intI* gene; a primary recombination site (*attI*); and an outward- orientated promoter (Pc) [163]. It has been found that class I integrons contribute to antimicrobial resistance in *Salmonella* isolates and *Stenotrophomonas maltophilia*, respectively [164,165]. A new rifampin resistance gene *arr-2*, located on a gene cassette within a class I integron, was found in *P. aeruginosa* [166]. Furthermore, the SOS response controls a series of genes responsible for DNA damage repair. A variety of studies has proved that the SOS response controls integron recombination and promotes antibiotic resistance development [167,168].

In addition, there might be a lot of genes (not only one or two genes) that contributes to the resistance mechanisms in bacteria. The conception of resistome was proposed by Gerard D. Wright for the collection of all the antibiotic resistance genes and their precursors in both pathogenic and non-pathogenic bacteria [169]. In the latest update, silent and proto-resistance genes were also included into the theoretical framework of the resistome [170]. This resistome would indirectly or directly induce the resistance mechanisms in bacteria. A comprehensive *P. aeruginosa* PA14 mutant library was constructed and screened for identifying genes involved in resistance to polymyxin B in this strain. Among the susceptible mutants, a significant number carried transposon insertions in lipopolysaccharide (LPS)-related genes (*galU*, *lptC*, *wapR*, and *ssg*). A decrease in the presence and/or inducibility of aminoarabinose-modified lipid A species provided an additional explanation for the supersusceptible phenotype of these mutants [171]. Complex ciprofloxacin resistome of more than 100 genes in *P. aeruginosa* PA14 was also screened using a mutant library [172]. In addition to polymyxin B and ciprofloxacin, the intrinsic resistome of *P. aeruginosa* to β-lactams was also investigated using a comprehensive library of transposon-tagged insertion mutants. It was found that 37 loci in the chromosome of *P. aeruginosa* contributed to its intrinsic resistance [173].

## 5. Conclusions

In summation, bacteria build three main lines of defense at spatially distinct locations to protect themselves against antimicrobial agents (Figure 1). Firstly, most bacteria will live in the form of biofilms to increase their resistance levels to antimicrobial compounds through specific resistance mechanisms, including restricted penetration of antimicrobial agents, alteration of metabolic rates and gene regulations and formation of persister cells. Secondly, antimicrobial agents have to enter cells via the cell wall and membrane to exert their activity. Mutations that result in alterations of the cell wall and membrane can render the cells resistant to an antibiotic. Meanwhile, the efflux pumps encased in the cell wall and membrane also contribute to the resistance of some antimicrobial agents by pumping them out of the bacterial cells. Thirdly, even when antimicrobial agents successfully get inside the bacterial cells, they still have to remain stable and accumulate at the target sites to inhibitory concentrations. In some situations, the antibiotic requires activation and must traverse to its target(s) in order to exert antimicrobial activity. Mutations that result in changes of target sites and gene expressions, and production of quenchers against the antimicrobial agents all enhance resistance levels. All respective resistance cases and some corresponding substances, proteins or genes mentioned in this review are also summarized in Table 1. For a given antimicrobial agent, it has to prevail against multiple lines of defense for successful antimicrobial action, where two or three lines of defense may work simultaneously. At the same time, for a given item in a defense line, its function may exhibit at other lines. For example, the encoded products by plasmids can play a role in both intracellular and extracellular cells. In addition, collective antibiotic resistance was also proposed in recent years. In any case, the three main bacterial lines of defense against antimicrobials theory categorized by their spatially distinct sites of action and distribution will help us to rapidly and clearly understand and conceptualize the predominant antibiotic resistance mechanisms in bacteria and design reasonable strategies to overcome these resistances.

**Table 1:** Summary of the main resistance mechanisms in three lines of defense for bacteria to combat antimicrobial agents in this review.

Line of Defense	Main Resistance Mechanisms	Related Substances, Proteins or Genes *	Representative References
The first	Reduced penetration of antimicrobial molecules	EPS	[32,33,34]
	Physiological gradients	-	[21,39,40]
	Formation of persister cells	-	[45,46]
	General stress response	*rpoS*, *anr*	[51,52]
The second	Cell wall	Peptidoglycan	[64,65]
	Cell membrane	Membrane proteins	[72,73,74]
	Action of efflux pumps	AcrAB/TolC, MexAB-oprM, MexCD-OprJ, MexEF-oprN, MexXY-oprM	[87,88,91,93,94,95,96,97,101,102,103]
The third	Ribosome and protein synthesis	RPPs	[107,109,111,112,113,114]
	Increasing the production of a metabolite	PABA	[5]
	Quorum sensing (QS) systems	LasR-LasI, RhlR-RhlI	[119,121,122]
	DNA synthesis	DNA gyrase, topoisomerase IV	[125,126]
	RNA synthesis	RNAP, rRNA methylases	[128,129,130]
	Plasmid mediated resistance	*ermC*, *cfr*, β-lactamase, *qnr*	[131,133,135,136,137,138,139,140,142,144]
	Mutations of the target gene in bacterial chromosome	*gyrA*, *parC*, *parE*, *marOR*, *acrR*	[148]
	Transposon	-	[153,154,155,156]
	Integrons	*intI*, *attI*, Pc, *arr-2*	[164,165,166]
	Resistome	-	[171,172,173]

* The listed cases represent only a part of examples in a given resistance mechanism, not for all.
